# Methylglyoxal as a Potential Mediator of Metabolic Memory in Diabetes-Related Complications: From Oxidative Stress to Macromolecular Glycation and Epigenetic Remodeling

**DOI:** 10.3390/antiox15101243

**Published:** 2026-09-26

**Authors:** Maya A. K. Stelzer, Abdul W. Khan, Jeffrey Kroon, Philippe Vangrieken, Nordin M. J. Hanssen

**Affiliations:** 1 Department of Vascular Medicine, Amsterdam UMC, Location AMC, Meibergdreef 9, 1105 AZ Amsterdam, The Netherlands; m.a.k.stelzer@amsterdamumc.nl; 2 Department of Experimental Vascular Medicine, Amsterdam UMC, Location AMC, Meibergdreef 9, 1105 AZ Amsterdam, The Netherlands; j.kroon@amsterdamumc.nl; 3 Laboratory Animal Research Center, Qatar University, Doha P.O. Box 2713, Qatar; wkhan@qu.edu.qa; 4 CARIM School for Cardiovascular Disease, Maastricht University, Universiteitssingel 50, 6229 ER Maastricht, The Netherlands; p.vangrieken@maastrichtuniversity.nl; 5 Department of Internal Medicine, Maastricht University Medical Center, Minderbroedersberg 4, 6211 LK Maastricht, The Netherlands; 6 Diabeter Centrum Amsterdam, Louwesweg 6, 1066 EC Amsterdam, The Netherlands

**Keywords:** methylglyoxal, oxidative stress, metabolic memory, diabetes-related complications, advanced glycation end-products, mitochondrial dysfunction, epigenetics

## Abstract

Diabetes-related complications often continue to progress despite improved glycemic control, a phenomenon known as metabolic memory. Increasing evidence suggests that methylglyoxal (MGO), a highly reactive dicarbonyl generated during glycolysis, may be an important mediator of this process. Hyperglycemia-induced oxidative stress promotes MGO formation, where MGO further promotes mitochondrial dysfunction and reactive oxygen species production, establishing a self-reinforcing cycle of stress. In addition, MGO modifies proteins, impairing their structure and function and promoting persistent inflammatory signaling through advanced glycation end-products. More recently, nucleic acids have emerged as important targets of dicarbonyl stress, with MGO-induced DNA and RNA glycation contributing to genomic instability and altered cellular function. Furthermore, MGO-mediated histone glycation and epigenetic remodeling may provide a mechanistic link between hyperglycemia and long-lasting changes in gene expression. These mechanisms are particularly relevant in diabetic kidney disease, where MGO contributes to inflammation, fibrosis and renal injury. However, direct evidence that MGO-induced molecular alterations and their functional consequences persist after normalization of both glucose and MGO remains limited. This review discusses the role of MGO in metabolic memory, focusing on oxidative stress, protein glycation, nucleic acid damage and epigenetic remodeling, and highlights emerging therapeutic strategies targeting dicarbonyl stress to prevent the progression of diabetes-related complications.

## 1. Introduction

Diabetes mellitus is a metabolic disorder that is characterized by persistent hyperglycemia, caused by impaired insulin secretion and/or reduced insulin sensitivity [1]. Chronic high blood glucose levels promote oxidative damage, endothelial dysfunction and inflammation, which in turn are associated with the development of diabetic kidney disease (DKD), retinopathy, neuropathy and cardiovascular disease [2]. Although intensive glycemic control reduces the risk of these diabetes-related complications [3], clinical studies have demonstrated that early periods of hyperglycemia can induce increased risk of vascular complications that persist even after normalization of blood glucose levels [4]. This phenomenon, commonly referred to as metabolic memory, denotes the persistence of an increased risk of complications after glycemia has been normalized. Two fundamentally different explanations must be distinguished: a durable molecular mark that outlives the initiating exposure, or a self-sustaining process that keeps the damaging exposure itself elevated [5]. The exact mechanisms underlying metabolic memory remain poorly defined, which limits the development of preventive strategies. Additionally, whether modern diabetes technologies can prevent the establishment of metabolic memory, and whether cardiorenal therapies can attenuate long-term vascular consequences, remains incompletely understood [5,6].

An important player in the development of diabetes-related complications is methylglyoxal (MGO), a highly reactive dicarbonyl compound that is primarily formed as a byproduct of glycolysis [7]. Under conditions with high glycolytic flux and oxidative stress, such as in diabetes, MGO is generated excessively [8]. Elevated plasma MGO levels have been associated with DKD, incident cardiovascular disease and mortality in individuals with type 1 (T1D) and type 2 diabetes (T2D), as well as with amputation and mortality in individuals with severe limb ischemia [9,10,11]. Traditionally, MGO has therefore been regarded primarily as a biomarker of diabetes and dicarbonyl stress [12]. However, because direct MGO concentrations are technically challenging to measure and reflect a dynamic and highly reactive pool, measurements of stable MGO-derived adducts are also frequently used to assess the biological consequences of dicarbonyl stress. Interestingly, emerging evidence suggests that MGO may also function as an active mediator of metabolic memory. Short periods of hyperglycemia, such as those occurring during an oral glucose tolerance test (OGTT), can transiently increase circulating MGO concentrations, particularly in individuals with impaired glucose metabolism or T2D [13]. However, whether smaller transient increases in MGO are sufficient to induce persistent molecular or functional changes remains unclear [13].

Despite increasing evidence linking MGO to diabetes-related complications, the mechanisms through which MGO could translate a transient exposure into a lasting one remain incompletely defined. One possibility requires no lasting molecular mark at all: MGO and oxidative stress may reinforce one another such that the exposure itself never normalizes. In particular, oxidative stress and MGO formation appear to constitute a positive feedback loop in which reactive oxygen species (ROS) enhance MGO generation, while MGO further impairs mitochondrial function and amplifies oxidative stress [14]. Distinguishing this from true molecular memory therefore requires evidence that alterations persist once both glucose and MGO have normalized.

A second class of mechanisms invokes durable modification of macromolecules, considered here in order of the turnover of the modified substrate. MGO non-enzymatically reacts with proteins, lipids and nucleic acids to form advanced glycation end-products (AGEs), which have been implicated in the progression of diabetes-related complications [2]. MGO-mediated glycation of proteins may contribute to long-lasting structural and functional alterations involved in vascular dysfunction and tissue remodeling [8]. Beyond protein glycation, MGO can also directly modify nucleic acids, including DNA and RNA, contributing to genomic instability and altered cellular function [15]. Finally, recent studies suggest that MGO may exert epigenetic effects through direct modification of histones and interference with canonical histone marks, thereby altering chromatin accessibility and transcriptional regulation [16,17]. Because epigenetic modifications can persist over time and regulate long-term gene expression patterns, MGO-induced epigenetic remodeling represents an important and underexplored mechanism behind metabolic memory. To establish MGO as a causal mediator of metabolic memory, it will be crucial to demonstrate that MGO-induced alterations persist after normalization of both glucose and MGO and that selective inhibition or removal of MGO prevents these effects.

In this review, we discuss the emerging role of MGO as a mediator of metabolic memory in diabetes-related complications. We specifically focus on the mechanistic axis linking oxidative stress, mitochondrial dysfunction and MGO generation and discuss how this pathway may contribute to the persistence of diabetes-related complications through protein modification, direct genomic damage and epigenetic remodeling (Figure 1). DKD is used throughout this review as a recurring example because the available clinical and experimental evidence for MGO is particularly extensive in the kidney. Extrapolation to retinopathy, neuropathy and macrovascular disease is therefore uncertain and is treated as such throughout. Traditionally, the formation of long-lived protein modifications was considered the major mechanism driving metabolic memory, but newer research also links MGO to direct modification of DNA/RNA and indirect modification of histone tails as additional routes to long-lasting adverse effects. In addition, we highlight potential therapeutic strategies targeting the ROS–MGO axis and discuss future perspectives for preventing long-term diabetes-related complications.

## 2. The ROS-MGO Axis: Oxidative Stress, Mitochondrial Dysfunction and MGO Generation

### 2.1. Oxidative Stress as a Driver of MGO Generation

Oxidative stress is widely recognized as a central driver of diabetes-related complications and has long been implicated in the development of metabolic memory [18]. In diabetes, chronic hyperglycemia increases intracellular glucose availability and enhances glycolytic flux. In susceptible cell types, this may increase electron pressure on the mitochondrial respiratory chain and promote mitochondrial ROS formation, particularly superoxide [14]. Hyperglycemia may additionally stimulate non-mitochondrial ROS-generating pathways and impair cellular antioxidant defenses. Sustained mitochondrial ROS production disrupts redox homeostasis and contributes to endothelial dysfunction, inflammation and tissue injury [19]. As a result, oxidative stress affects multiple downstream processes involved in the progression of diabetes-related complications. Importantly, oxidative stress also induces metabolic alterations that cause the generation and accumulation of reactive dicarbonyl compounds, including MGO [20].

MGO is primarily generated through the spontaneous degradation of the glycolytic intermediates glyceraldehyde-3-phosphate and dihydroxyacetone phosphate [8]. Under normal conditions, these phosphates are regulated by glycolytic enzymes. However, oxidative stress impairs several of these enzymes, including GAPDH and mitochondrial complex III proteins, resulting in the accumulation of upstream glycolytic intermediates and enhanced MGO formation [20,21]. Furthermore, intracellular MGO levels are normally tightly controlled by the glyoxalase (GLO) system. MGO first reacts spontaneously with glutathione to form a hemithioacetal, which is converted by GLO1 into S-D-lactoylglutathione and subsequently hydrolyzed by GLO2 to D-lactate, thereby regenerating glutathione. This detoxification pathway is thus dependent on intracellular glutathione [7]. Additional enzymes, including members of the aldo-keto reductase family, can also contribute to MGO detoxification, although their relative contribution in vivo remains unknown [22]. Since oxidative stress depletes intracellular glutathione, it impairs GLO activity and reduces the cellular capacity to eliminate MGO, thereby promoting dicarbonyl stress [20]. In the context of diabetes, MGO is therefore not only a byproduct of elevated glucose levels, but also a consequence of metabolic and mitochondrial dysregulation induced by oxidative stress. The direction of this relationship is, however, difficult to resolve experimentally: most studies manipulate one arm and measure the other during continuous exposure, so the relative contribution of oxidative stress to MGO accumulation, and of MGO to oxidative stress, remains uncertain.

Additionally, direct measurement of MGO in biological samples is technically challenging because of its high reactivity and rapid conversion into protein and nucleic acid adducts. Quantification of free MGO generally requires rapid stabilization and derivatization, followed by chromatographic separation and detection, with stable-isotope dilution LC-MS/MS providing high analytical specificity [23]. Pre-analytical conditions are particularly important because MGO can be generated or lost during sample processing, potentially leading to changes in measured concentrations. Moreover, circulating MGO represents only one component of total dicarbonyl exposure and may not reflect intracellular or tissue-specific MGO concentrations. Measurements of MGO-derived adducts, such as methylglyoxal-derived hydroimidazolone (MG-H1) in proteins and N2-(1-carboxyethyl)-2′-deoxyguanosine (CEdG) in nucleic acids, can therefore provide complementary molecular footprints of dicarbonyl stress [24,25]. These adducts should not, however, be interpreted as direct measurements of free MGO.

### 2.2. MGO-Induced Mitochondrial Dysfunction and ROS Production

Interestingly, increasing evidence suggests that MGO itself further promotes mitochondrial dysfunction and oxidative stress, thereby establishing a positive feedback loop [8,26]. MGO can modify proteins involved in oxidative phosphorylation, including components of the electron transport chain, resulting in impaired mitochondrial respiration and enhanced mitochondrial ROS production [24]. In addition, MGO-mediated glycation of mitochondrial membrane proteins disrupts membrane integrity, further compromising mitochondrial function and resulting in increased oxidative stress [26,27].

In addition to its direct effects on mitochondrial proteins, MGO disrupts intracellular redox homeostasis by indirectly affecting mitochondrial function and ROS production. Liu et al. demonstrated that intracellular accumulation of MGO in pancreatic β-cells inhibits thioredoxin 2 (Trx2), a key mitochondrial antioxidant protein, resulting in excessive mitochondrial ROS production, overproduction of AGEs and insulin secretion paralysis [26]. Furthermore, a study on the effect of MGO on human epithelial kidney cells showed that MGO-derived AGEs induced mitochondrial dysfunction and endoplasmic reticulum stress, ultimately promoting apoptosis [27]. Together, these studies indicate that MGO is not only generated as a consequence of oxidative stress but may also amplify oxidative damage by disrupting mitochondrial function and cellular antioxidant defenses. However, the direct evidence for MGO-induced mitochondrial dysfunction remains largely based on experimental models, including cultured pancreatic β-cells and renal epithelial cells, while in vivo evidence is more limited and has primarily been demonstrated for MGO-derived AGEs rather than MGO itself.

### 2.3. The ROS-MGO Axis in Metabolic Memory and DKD

Together, these findings highlight the ROS-MGO axis as a key mechanism underlying the development and progression of diabetes-related complications. Through its relationship with oxidative stress and mitochondrial dysfunction, MGO may contribute to persistent cellular dysfunction after glycemic normalization, thereby providing a plausible mechanistic basis for metabolic memory. However, most experimental studies have assessed cellular dysfunction during ongoing MGO exposure rather than after normalization of both glucose and MGO [8]. Therefore, a better understanding of the role of MGO in this process may provide insights into why and how diabetes-related complications, including DKD, continue to progress despite improved blood glucose levels. The relationship between MGO and DKD may be more complex than initially thought. Experimental studies have previously demonstrated that increased MGO levels promote renal oxidative stress, inflammation and fibrosis, thereby contributing to the development and progression of DKD [20]. However, recent studies suggest that this may be a two-way relationship. As renal function declines, the kidney becomes less effective at detoxifying reactive dicarbonyl compounds and their glycation products, thus promoting MGO accumulation and stress [28]. In support of this, Jensen et al. demonstrated that higher plasma MGO levels were associated with a greater decline in estimated glomerular filtration rate (eGFR) and the development of albuminuria in individuals with T2D [29]. Consistent with these findings, Hanssen et al. reported that both fasting and post-OGTT plasma MGO concentrations were associated with markers of DKD in individuals with and without prediabetes or T2D [30]. These findings support the concept of a positive feedback loop in which MGO contributes to the development of DKD, while renal dysfunction promotes the accumulation of MGO. These observations indicate that MGO should not only be seen as a driver of DKD, but also as part of a dynamic network in which kidney dysfunction sustains dicarbonyl stress.

## 3. Protein Modifications and AGE Formation: A Mechanism of Metabolic Memory?

### 3.1. MGO-Mediated Protein Modification and Dysfunction

Protein glycation is one of the best-characterized pathological consequences of MGO accumulation and has long been implicated in the development of diabetes-related complications. Unlike enzymatic post-translational modifications, glycation occurs through a non-enzymatic reaction between reactive carbonyl compounds and amino acid residues. Due to its high electrophilic reactivity, MGO preferentially reacts with arginine residues and, to a lesser extent, lysine and cysteine residues [8]. This results in the formation of several stable AGEs, of which MG-H1 is the most abundant and quantitatively important MGO-derived protein modification in vivo. Other MGO-derived adducts, including Nε-(carboxyethyl)-lysine (CEL) and argpyrimidine (ArgPyr), can also be detected in biological tissues and fluids, but are generally less abundant [24]. Protein glycation by MGO affects a wide range of structural, enzymatic and signaling proteins, thus disrupting normal cellular function. Glycation can alter protein conformation, reduce enzymatic activity, impair protein–protein interactions and alter recognition and processing by cellular protein quality-control systems [2]. Moderate glycation-induced protein unfolding may facilitate proteasomal degradation, whereas extensive AGE formation and intra- or intermolecular cross-linking can reduce susceptibility to proteolytic degradation [31]. Importantly, AGE formation is often irreversible and thus these modifications may persist long after normalization of blood glucose levels. In that context, MGO-induced protein glycation provides a potential molecular basis for metabolic memory.

Several studies have demonstrated that MGO-mediated protein glycation affects a wide range of proteins involved in essential cellular processes. Proteomic analyses have identified MGO-modified proteins involved in glycolysis, protein folding, mitochondrial function and cellular stress response, highlighting the broad impact of dicarbonyl stress on cellular function [24]. MGO preferentially modifies functionally important arginine residues within these proteins, making them more susceptible to misfolding and functional inactivation [24]. In addition, Chen et al. demonstrated that MGO induces protein cross-links, which further alter protein stability and function [32]. Numerous MGO-induced cross-linking sites were identified across proteins involved in metabolic pathways and RNA processing, suggesting that dicarbonyl stress disrupts protein function not only through individual glycation, but also by altering protein–protein interactions [32]. Furthermore, a study by Donnellan et al. found that glycolytic enzymes are particularly susceptible to MGO modification [33]. These modifications occurred at functionally important residues, including active-site residues, suggesting that they may alter enzyme activity and consequently affect glycolytic flux [33].

### 3.2. AGE-RAGE Signaling

Beyond its direct effects on protein structure and function, MGO-derived AGEs affect the cell through activation of the receptor for AGEs (RAGE). Binding of AGEs to RAGE activates several intracellular signaling pathways, including Nuclear Factor kappa B (NF-кB), resulting in the production and secretion of pro-inflammatory cytokines (Figure 2) [34]. Importantly, direct MGO-mediated protein modification and AGE–RAGE signaling represent distinct mechanisms. Not all MGO-derived AGE structures activate RAGE to the same extent, and RAGE has multiple ligands [20,35]. The biological consequences of MGO therefore cannot be attributed exclusively to AGE–RAGE signaling and may also result from direct structural and functional modification of proteins independently of RAGE. Persistent activation of RAGE also increases ROS generation, oxidative stress and apoptosis, resulting in tissue injury [20]. Because some AGEs can persist within tissues for prolonged periods, they may contribute to sustained RAGE signaling beyond periods of hyperglycemia, contributing to sustained inflammation and fibrotic remodeling. The extent to which this persistence contributes to metabolic memory, however, remains incompletely established.

### 3.3. The Persistence and Clearance of Glycated Proteins

Whether MGO-mediated glycation can act as a molecular memory depends primarily on how long the modified protein remains present in the cell. One determinant is a decrease in the clearance of glycated proteins. Cells have two main processes for degrading intracellular proteins: the ubiquitin–proteasome system and autophagy [36]. However, as discussed previously, MGO-mediated glycation alters protein structure and promotes the formation of cross-links, both of which make proteins more resistant to proteolytic degradation [31]. Consequently, AGEs accumulate within cells, where they continue to impair cellular function and promote oxidative stress. This is particularly relevant for long-lived proteins, whose slow turnover allows MGO-derived glycation to accumulate and persist, thereby prolonging their effects [37]. In addition to intracellular degradation, circulating AGEs are removed through receptor-mediated uptake and renal clearance [37]. However, in the case of metabolic stress, renal function declines, leading to impaired AGE clearance. The accumulation of AGEs in the kidneys in turn promotes extracellular matrix (ECM) remodeling, inflammation and fibrosis, which further stimulate renal injury [34]. These observations suggest that once renal injury is established, impaired AGE clearance may further enhance dicarbonyl stress, creating a self-reinforcing cycle that contributes to progressive kidney dysfunction.

### 3.4. MGO-Mediated ECM Glycation and Renal Injury

In line with this, several studies have demonstrated that MGO modifies proteins involved in pathways that are directly relevant to diabetes-related complications. One well-characterized target is the ECM. MGO-mediated glycation of ECM proteins, like collagen IV and laminin, results in altered cell adhesion, increased matrix stiffness and disrupted cell–matrix interactions [20,34]. This then promotes endothelial dysfunction and fibrosis, which contributes to tissue remodeling in diabetes-related complications such as DKD. Consistent with this, MGO-induced AGE formation and AGE–collagen cross-linking have been demonstrated experimentally, while anti-glycation treatment reduced MGO-induced renal cellular damage [38]. Similarly, experimental studies have shown that inhibition of AGE formation or enhancement of MGO detoxification attenuates renal fibrosis, inflammation and albuminuria, supporting a causal role for MGO-mediated protein glycation in the development of DKD [39]. Because ECM proteins have a slow turnover, MGO-induced modifications may remain present for prolonged periods and continue to promote renal injury despite improvements in glycemic control. These observations support the possibility that persistent protein glycation by MGO contributes to the development and progression of DKD and may represent an important mechanism underlying metabolic memory.

## 4. MGO-Induced DNA and RNA Modifications

### 4.1. The Effects of MGO-Mediated Glycation of DNA

Although the pathological effects of MGO have traditionally been attributed to protein glycation and AGE formation, accumulating evidence suggests that nucleic acids are also important targets of dicarbonyl stress [15]. In DNA, MGO preferentially reacts with guanine residues, resulting in the formation of stable DNA glycation adducts [40]. Among these, CEdG is one of the most abundant and extensively studied DNA glycation products (Figure 3). Interestingly, elevated levels of CEdG have been detected in experimental models of diabetes and in tissues exposed to increased MGO concentrations [41]. These findings establish that DNA glycation occurs under conditions of increased dicarbonyl stress, but do not by themselves demonstrate that CEdG formed during transient hyperglycemia persists after normalization of glucose and MGO. Although DNA glycation products may be relatively stable, direct evidence that CEdG generated during a transient metabolic insult remains present and functionally relevant after normalization of both glucose and MGO is currently limited. In that regard, CEdG formation by MGO represents a plausible but not yet established candidate mediator of metabolic memory.

The formation of CEdG is particularly relevant because it directly affects genomic integrity and, therefore, could potentially translate short periods of hyperglycemia into longer-lasting alterations. The modification of deoxyguanosine (dG) by MGO alters the chemical structure of guanine and disrupts normal base pairing, increasing the likelihood of replication errors and point mutations [42]. Various studies have demonstrated that CEdG is mutagenic and promotes base substitutions, including G-T and G-C transversions [41,42]. The importance of these findings is further supported by studies demonstrating associations between CEdG levels, hyperglycemia and DKD [43]. However, evidence that these effects persist after removal of the initiating MGO exposure remains limited.

In addition to the formation of CEdG, MGO-mediated DNA damage may have broader consequences for cellular homeostasis. Accumulation of DNA glycation adducts induces replication stress and activates DNA damage response pathways, resulting in cellular dysfunction [44,45]. A study by Liu et al. on the effect of MGO on human embryonic kidney cells confirmed these findings. An increase in ROS levels, activation of the pro-inflammatory NF-кB signaling pathway, an increase in oxidative damage and double-strand break markers and activation of the Ataxia–Telangiectasia Mutated (ATM) pathway were observed in the cells after MGO treatment [45]. The link between MGO exposure and the ATM pathway, which is a DNA damage response pathway, is of particular interest, since prolonged DNA damage signaling has been associated with the induction of cellular senescence [46]. Importantly, these studies did not directly establish a senescent phenotype. Therefore, a role for cellular senescence in MGO-induced metabolic memory remains based on the activation of DNA damage-response pathways rather than a directly demonstrated consequence of MGO exposure. Cellular senescence is a state in which cells permanently stop dividing, characterized by an increase in pro-inflammatory signaling [46]. This is important in the context of diabetes, since senescence of pancreatic β-cells impairs insulin secretion and senescent cells in organs can contribute to the development of diabetes-related complications [47]. Senescence is of particular conceptual interest for metabolic memory, since it constitutes a stable cellular state that is maintained by pro-inflammatory signaling rather than by the persistence of any individual adduct. Once established, such a state may outlast both the glycemic insult and the MGO exposure that initiated it, and may be amenable to intervention independently of MGO itself.

### 4.2. Oxidative and Dicarbonyl Stress as Drivers of mtDNA Damage

Although MGO is capable of altering nuclear DNA (nDNA), mitochondrial DNA (mtDNA) seems to be particularly susceptible to DNA damage. A study by Yakes et al. showed that mtDNA is a critical cellular target for ROS and accumulates significantly more oxidative damage under stress conditions than nDNA [48]. Mitochondria possess active DNA repair mechanisms, particularly base-excision repair, but have a more limited repair repertoire than the nucleus [49]. Consequently, DNA damage induced by oxidative and dicarbonyl stress is more likely to accumulate within the mitochondrial genome. Although oxidative mtDNA damage is well established, direct evidence for preferential or persistent MGO-mediated glycation of mtDNA remains limited [49]. Several studies have demonstrated that MGO impairs mitochondrial function through modification of mitochondrial proteins involved in oxidative phosphorylation, resulting in reduced ATP production and enhanced ROS generation [8,24,26]. Thus, while MGO-mediated mtDNA damage may contribute to mitochondrial dysfunction, this possibility remains insufficiently investigated. Nevertheless, MGO-induced mitochondrial protein damage and the resulting increase in ROS may further exacerbate oxidative stress, potentially reinforcing the ROS–MGO cycle described above.

### 4.3. RNA Glycation and Its Effects on Cellular Function

In addition to DNA, RNA has emerged as an important target of MGO-mediated glycation. Similar to its interaction with DNA, MGO preferentially reacts with guanosine residues in RNA, resulting in the formation of glycation adducts, including N^2^-(1-carboxyethyl)-guanosine (CEG) (Figure 3) [50]. Since RNA molecules are directly involved in protein synthesis and gene regulation, MGO-induced RNA modifications potentially play an important role in disease progression. MGO-derived RNA adducts reduce RNA stability and impair translation efficiency, leading to decreased protein synthesis and altered cellular function [50]. Additionally, an association between RNA glycation products, metabolic stress and diabetes-related complications has been observed [50]. In support of this, Shuck et al. detected elevated levels of MGO-derived RNA adducts in individuals with presymptomatic T1D [51]. This suggests that RNA glycation occurs early during disease development and thus may play a role in T1D development. Because many RNA molecules have relatively short lifetimes, direct persistence of RNA glycation within RNA molecules is unlikely to represent a durable molecular mechanism of metabolic memory. However, RNA glycation could act as an initiating or amplifying factor that contributes to more persistent downstream changes in proteostasis, cellular phenotype or gene regulation [50]. In addition, modified ribonucleotides could theoretically persist within the cellular ribonucleotide triphosphate (rNTP) pool. Intracellular rNTP concentrations can exceed deoxyribonucleoside triphosphate (dNTP) concentrations, providing a comparatively large pool in which modified ribonucleotides could potentially accumulate [52]. If CEG-modified ribonucleotides are not efficiently removed, their persistence could allow subsequent incorporation into newly synthesized RNA. This mechanism remains hypothetical, and direct evidence for persistence and reincorporation of CEG-modified ribonucleotides is currently lacking.

Together, these findings suggest that MGO affects nucleic acids at multiple levels. Through the formation of DNA and RNA adducts, induction of DNA damage responses, potential promotion of cellular senescence and modification of RNA stability, MGO may alter cellular function. Importantly, some downstream consequences of these processes may persist after normalization of blood glucose levels, making them plausible contributors to metabolic memory and diabetes-related complications.

## 5. Epigenetic Remodeling by MGO

Epigenetic mechanisms play a central role in the regulation of gene expression and cellular phenotypes. Unlike genetic alterations, epigenetic modifications influence gene expression without altering the underlying DNA sequence. Epigenetic regulation encompasses heritable and reversible changes in gene expression. Major epigenetic mechanisms include DNA methylation, histone modifications, chromatin remodeling, and non-coding RNAs (ncRNAs). Histone proteins package DNA into nucleosomes and undergo diverse post-translational modifications, including acetylation, methylation, phosphorylation, ubiquitination, and SUMOylation, which regulate chromatin accessibility and transcriptional activity [53]. In addition, ATP-dependent chromatin-remodeling complexes alter nucleosome positioning and higher-order chromatin organization in response to environmental and metabolic cues [54]. Together, these interconnected epigenetic processes provide a molecular framework through which hyperglycemia can induce persistent changes in gene expression, contributing to metabolic memory and the progression of diabetic complications. Crucially, epigenetic modifications can persist long after the initiating stimulus has disappeared, highlighting them as a potential underlying mechanism of metabolic memory [18]. In support of this, hyperglycemia has been shown to induce epigenetic alterations in endothelial cells that persist even after glycemic normalization [16]. Importantly, these studies demonstrate persistence of hyperglycemia-induced epigenetic alterations rather than persistence of MGO-induced modifications specifically. Therefore, the available evidence supports a broader role for metabolic stress in epigenetic memory, while the contribution of MGO remains less directly established. Whether MGO contributes to epigenetic remodeling is therefore examined below along two lines of evidence: direct glycation of histone tails, and modification of the enzymes that establish and remove canonical histone marks.

### 5.1. MGO-Mediated Histone Glycation

Unlike conventional post-translational histone modifications, such as methylation and acetylation, MGO can directly react with lysine and arginine residues within histone tails, resulting in AGE formation [55]. Histone tails are crucial in chromatin accessibility, since they serve as binding platforms for chromatin-modifying enzymes and transcriptional regulators. Consequently, MGO-mediated histone glycation has the potential to disrupt chromatin organization and normal gene regulation. Galligan et al. demonstrated that MGO-derived modifications were present on all four core histones and that histone glycation accumulated under increased glycolytic flux [17]. Interestingly, histone glycation by MGO has been observed on many of the same lysine and arginine residues that are targeted by canonical epigenetic marks, suggesting that MGO interferes with normal epigenetic regulatory mechanisms and changes gene expression patterns [17]. These findings establish histones as biologically relevant targets of dicarbonyl stress and provide a link between cellular metabolism and chromatin regulation. Histone glycation may not necessarily be permanent. DJ-1, for example, has been reported to function as a deglycase capable of removing MGO-derived modifications from glycated proteins [55]. However, this proposed deglycase activity remains controversial. Other studies have suggested that the observed activity can instead be explained by glyoxalase activity, in which DJ-1 detoxifies free MGO rather than directly repairing pre-existing MGO-derived protein adducts [56,57]. Thus, whether DJ-1 directly removes MGO-derived modifications from histones in vivo remains unresolved. The persistence of these modifications may therefore depend on the balance between ongoing glycation, MGO detoxification, potential enzymatic repair, proteolytic turnover and replacement of modified histones [55,58].

### 5.2. The Role of MGO in Epigenetic Regulation: Epigenetic Enzymes

In addition to direct modification of histones, MGO may also affect the activity of epigenetic enzymes. Several studies have shown that oxidative and dicarbonyl stress influence the expression and activity of epigenetic regulators such as histone methyltransferases. A study by El-Osta et al. demonstrated that hyperglycemia resulted in an increase in ROS production, MGO formation and the activation of the NF-кB pathway [16]. Importantly, this inflammatory response was caused by increased methylation of histone 3 lysine 4 (H3K4) at the NF-кB subunit p65 promoter through recruitment of the methyltransferase Set7. These epigenetic changes were sustained after normalization of blood glucose levels and experimental manipulation of MGO formation supported a contribution of MGO to these changes [16]. However, this study did not demonstrate that MGO-mediated histone glycation itself constitutes the persistent epigenetic mark. Instead, the findings suggest that chronic activation of the ROS–MGO axis promotes epigenetic remodeling through both direct and indirect mechanisms (Figure 4).

### 5.3. From Epigenetic Remodeling to DKD

The clinical relevance of these observations is supported by studies demonstrating persistent epigenetic alterations in individuals with T1D. Epigenetic analyses revealed differences in DNA methylation and histone acetylation patterns associated with prior hyperglycemia, supporting the concept of metabolic memory in T1D [58,59]. Interestingly, several of these methylation changes were linked to the future development of diabetes-related complications. Although these studies provide evidence for epigenetic metabolic memory, they do not establish MGO as the direct cause of the observed epigenetic alterations. The potential relevance of MGO-induced epigenetic remodeling, however, is further evident in DKD. Increasing evidence suggests that persistent epigenetic alterations contribute to the progression of this disease by maintaining the expression of genes involved in inflammation, oxidative stress and fibrosis [60,61]. Indeed, changes in DNA methylation and histone modifications have been identified in cultured podocytes, mesangial cells and tubular epithelial cells exposed to high glucose, as well as in renal tissue from diabetic animal models and individuals with DKD [61,62]. These epigenetic modifications have been linked to sustained activation of the NF-кB and TGF-β pathways, which in turn are associated with inflammation and fibrosis.

Collectively, these findings support a role for MGO in epigenetic remodeling through both direct and indirect mechanisms. Direct glycation of histone proteins may alter chromatin structure and interfere with canonical epigenetic marks, while activation of the ROS–MGO axis promotes oxidative stress-dependent chromatin remodeling and persistent changes in gene expression. However, the persistence of MGO-derived histone glycation itself after normalization of glucose and MGO remains insufficiently demonstrated. Acting together with protein glycation, DNA damage and mitochondrial dysfunction, these epigenetic alterations may translate transient metabolic stress into long-lasting cellular reprogramming. Consequently, MGO-induced epigenetic remodeling represents a plausible mechanism linking short episodes of hyperglycemia to metabolic memory and the continued progression of diabetes-related complications, particularly DKD.

## 6. Therapeutic Avenues and Future Perspectives

### 6.1. Treatment Beyond Glycemic Control: Targeting MGO

Current treatment options for diabetes include glycemic management as well as therapies that provide cardiovascular and kidney protection. SGLT2 inhibitors, such as dapagliflozin and empagliflozin, GLP-1 receptor agonists, such as semaglutide and liraglutide, and automated insulin delivery systems have improved glycemic and cardiorenal outcomes [63,64,65]. However, it remains unclear whether these therapies can prevent or reverse established metabolic memory. Intensive glycemic control has been shown to reduce the risk of diabetes-related complications in both T1D and T2D [3]. Nevertheless, several studies have demonstrated that these complications continue to develop and progress despite improved glycemic control, suggesting that normalization of blood glucose levels is not sufficient to completely prevent long-term complications [4,5]. This observation is particularly relevant in the concept of metabolic memory and highlights the need for therapeutic strategies that target the molecular consequences of hyperglycemia, instead of hyperglycemia itself.

Increasing evidence suggests that dicarbonyl stress, driven by MGO, may be a promising therapeutic target for preventing diabetes-related complications. Although MGO generation is closely connected to chronic hyperglycemia, recent findings indicate that transient elevations in glucose may also contribute to dicarbonyl stress. Maessen et al. demonstrated that plasma dicarbonyl concentrations, including MGO, increased significantly following an OGTT, with the greatest increases observed in individuals with impaired glucose metabolism and T2D [13]. Similarly, a recent study showed that 2 h post-load glucose was associated with MGO concentrations and that MGO partly mediated the association between post-load glucose and inflammation [66]. In addition, Hanssen et al. showed that both fasting and post-OGTT plasma MGO concentrations were associated with DKD and other microvascular complications in individuals with and without T2D [30]. Together, these findings suggest that short-term glucose spikes may contribute to MGO accumulation and its downstream effects, in addition to sustained hyperglycemia. Because MGO-induced protein modifications and their downstream consequences can persist beyond normalization of blood glucose levels, therapeutic strategies that directly reduce dicarbonyl stress may provide benefits by limiting the molecular processes that drive metabolic memory. However, evidence that targeting MGO prevents or reverses metabolic memory remains predominantly experimental and has not yet been established in clinical intervention studies.

### 6.2. Dietary Interventions to Reduce Endogenous MGO Formation

Dietary strategies that reduce postprandial glucose excursions, including carbohydrate restriction or intermittent fasting, may represent one approach to decrease endogenous MGO formation [13,66]. Indeed, short-term energy restriction has been shown to reduce postprandial dicarbonyl stress in individuals with T2D [67]. However, whether such reductions are sufficient to prevent the persistent molecular alterations associated with metabolic memory remains unknown. Moreover, not all dietary interventions are expected to decrease MGO formation. For example, nutritional ketosis has been associated with increased circulating MGO concentrations, indicating that carbohydrate restriction does not necessarily reduce dicarbonyl stress [68]. Importantly, endogenous MGO formation should be distinguished from dietary MGO exposure. Although endogenously generated MGO has consistently been linked to diabetic complications, prospective epidemiological studies have reported that higher habitual dietary MGO intake is associated with a lower incidence of T2D and cardiovascular disease [69,70]. These observations suggest that the biological effects of dietary MGO differ fundamentally from those of endogenously generated MGO and emphasize that therapeutic strategies should primarily target endogenous dicarbonyl stress.

### 6.3. Scavenging MGO and Limiting AGE Formation

Several therapies aimed at limiting MGO accumulation and its downstream effects have been developed. Some of these approaches focus on the scavenging of reactive dicarbonyl compounds before AGE formation occurs. One of the first compounds that was researched in this context is the AGE inhibitor aminoguanidine. This compound was highly promising, specifically for preventing DKD and cardiovascular disease, since it reduced AGE formation, cross-linking, ROS production and albuminuria in animal models [71,72]. However, clinical development of aminoguanidine was limited by an unfavorable side-effect profile [72]. Similarly, pyridoxamine, a vitamin B6 derivative, has been shown to scavenge dicarbonyls and inhibit dicarbonyl-mediated glycation [73]. Additionally, it normalized albuminuria and lowered AGE levels as well as preventing renal disease in diabetic animal models [39,71]. Unfortunately, further trials failed to demonstrate consistent improvements in renal function in individuals with diabetes, possibly reflecting differences in disease stage, dosing, patient selection or trial design [73]. Finally, carnosine is an endogenous carbonyl scavenger that has been researched for its therapeutic potential [74]. Carnosine reacts with MGO and, in that way, protects proteins against glycation [74]. In addition, a study by Liu et al. reported that carnosine reduced inflammation, fibrosis and renal injury in a diabetic mouse model [75]. Unfortunately, carnosine is degraded by the enzyme carnosinase, resulting in a relatively short half-life in humans. Overall, these findings provide preclinical support for dicarbonyl scavenging, but clinical evidence for prevention of diabetes-related complications remains limited.

### 6.4. Enhancing Endogenous MGO Detoxification

Current therapeutic avenues focus not only on scavenging MGO, but also on enhancing endogenous detoxification mechanisms of dicarbonyl stress. The glyoxalase system, and particularly GLO1, represents a highly specific target in this context, as it is the primary pathway responsible for the intracellular detoxification of MGO [7]. Increasing GLO1 activity could therefore provide a direct means of reducing MGO accumulation and limiting its downstream effects. Indeed, GLO1 inducers have been shown to decrease AGE production, inflammation and renal injury [76]. These findings are particularly relevant to the concept of MGO as a targetable mediator of diabetes-related complications, as they suggest that increasing endogenous MGO detoxification can be translated into protection against downstream tissue damage. In a study by Xue et al. an increase in GLO1 expression by activation of the nuclear factor erythroid 2-related factor 2 (Nrf2), led to improved cellular resistance to dicarbonyl stress [77]. Additionally, renal GLO1 levels decreased, while protein and nucleotide glycation increased in *Nrf2^−/−^* mice [77]. Together, these findings suggest that enhancing endogenous MGO detoxification may be a promising strategy to directly target a key upstream mediator of diabetes-related tissue injury, including DKD. Importantly, however, GLO1 expression alone may not fully determine MGO detoxification capacity, as its activity depends on glutathione availability and may be complemented by other detoxification pathways. Future studies should therefore determine whether sustained enhancement of GLO1 activity can reduce MGO accumulation in vivo and whether this translates into prevention or reversal of the persistent molecular changes associated with metabolic memory.

### 6.5. Targeting Epigenetics: Opportunities and Challenges

Lastly, the epigenetic mechanisms that underlie metabolic memory may be interesting therapeutic targets. These approaches target downstream epigenetic persistence rather than MGO itself. Because epigenetic alterations can persist long after normalization of blood glucose levels, therapies aimed at reversing these changes have the potential to decrease the inflammatory and fibrotic signaling that drives diabetes-related complications [61]. Importantly, targeting these downstream epigenetic pathways would not directly remove MGO-derived glycation adducts, but may instead counteract the persistent chromatin and transcriptional changes that arise downstream of, or alongside, MGO-induced epigenetic remodeling. One epigenetic regulator that is currently researched in the context of diabetes-related complications is enhancer of zeste homolog 2 (EZH2), a histone 3 lysine 27 (H3K27) methyltransferase [78]. EZH2 catalyzes trimethylation of H3K27, resulting in transcriptional repression of genes involved in inflammation, fibrosis and cellular differentiation. Dysregulated EZH2 activity has been implicated in several kidney diseases, including DKD, making it an attractive target for therapeutic intervention [79]. However, therapeutic targeting of EZH2 remains challenging, since its function is highly cell type and context-dependent. Aziz et al. demonstrated that inhibition of EZH2 reduced endothelial-to-mesenchymal transition and vascular injury in diabetic mice [78]. In contrast, Siddiqi et al. showed that, in diabetic rats, EZH2 protects podocytes from oxidative stress and renal injury [80]. Together, these findings show that successful epigenetic therapies will require precise, cell-specific modulation of epigenetic regulators. Nevertheless, targeting downstream epigenetic mechanisms may provide an opportunity to interfere with the persistent molecular consequences of metabolic stress, making this a promising avenue for the prevention of diabetes-related complications, such as DKD. These therapeutic observations are currently based largely on experimental models and their relevance to MGO-dependent metabolic memory in humans remains to be established.

Furthermore, advances in epigenomic technologies have enabled large-scale characterization of epigenetic variation across populations and tissues, revealing widespread associations with cardiometabolic traits, inflammation, and vascular dysfunction [81,82]. However, despite this rapid progress, translation into clinical practice has been slow and thus the enthusiasm for epigenetic applications often exceeds the available clinical evidence, with limited validation in prospective or interventional settings [81].

### 6.6. Limitations and Future Perspectives

Several limitations currently prevent firm conclusions about MGO as a causal mediator of metabolic memory. Firstly, plasma MGO may not reflect intracellular or tissue-specific exposure. Free MGO is highly reactive and rapidly converted into protein and nucleotide adducts, while MGO concentrations can also be affected by sample collection, processing and storage. Thus, direct plasma measurements and stable MGO-derived adducts provide complementary but distinct information about dicarbonyl stress [25]. Additionally, many experimental studies use sustained or supraphysiological MGO concentrations. Future studies should therefore model transient, physiologically relevant MGO elevations, include follow-up after normalization of glucose and MGO, and determine whether selective MGO lowering prevents or reverses persistent functional abnormalities.

Future therapeutic strategies will likely require a combination of improved glycemic control and interventions that reduce dicarbonyl stress or limit its long-lasting downstream effects. However, establishing a causal role for MGO in metabolic memory will require studies demonstrating that transient MGO exposure induces persistent molecular and functional alterations that remain after normalization of MGO levels and that selective inhibition of MGO formation or activity prevents these changes. Elucidating the molecular mechanisms linking MGO to oxidative stress, protein glycation, genomic instability and epigenetic remodeling will be essential for the development of targeted therapies aimed at interrupting metabolic memory. Such approaches may ultimately complement conventional glucose-lowering therapies and provide new opportunities to delay or prevent the progression of DKD and other diabetes-related complications. At present, however, the available evidence supports MGO as a plausible and potentially important mediator of metabolic memory rather than as an established causal mediator.

## Figures and Tables

**Figure 1:**
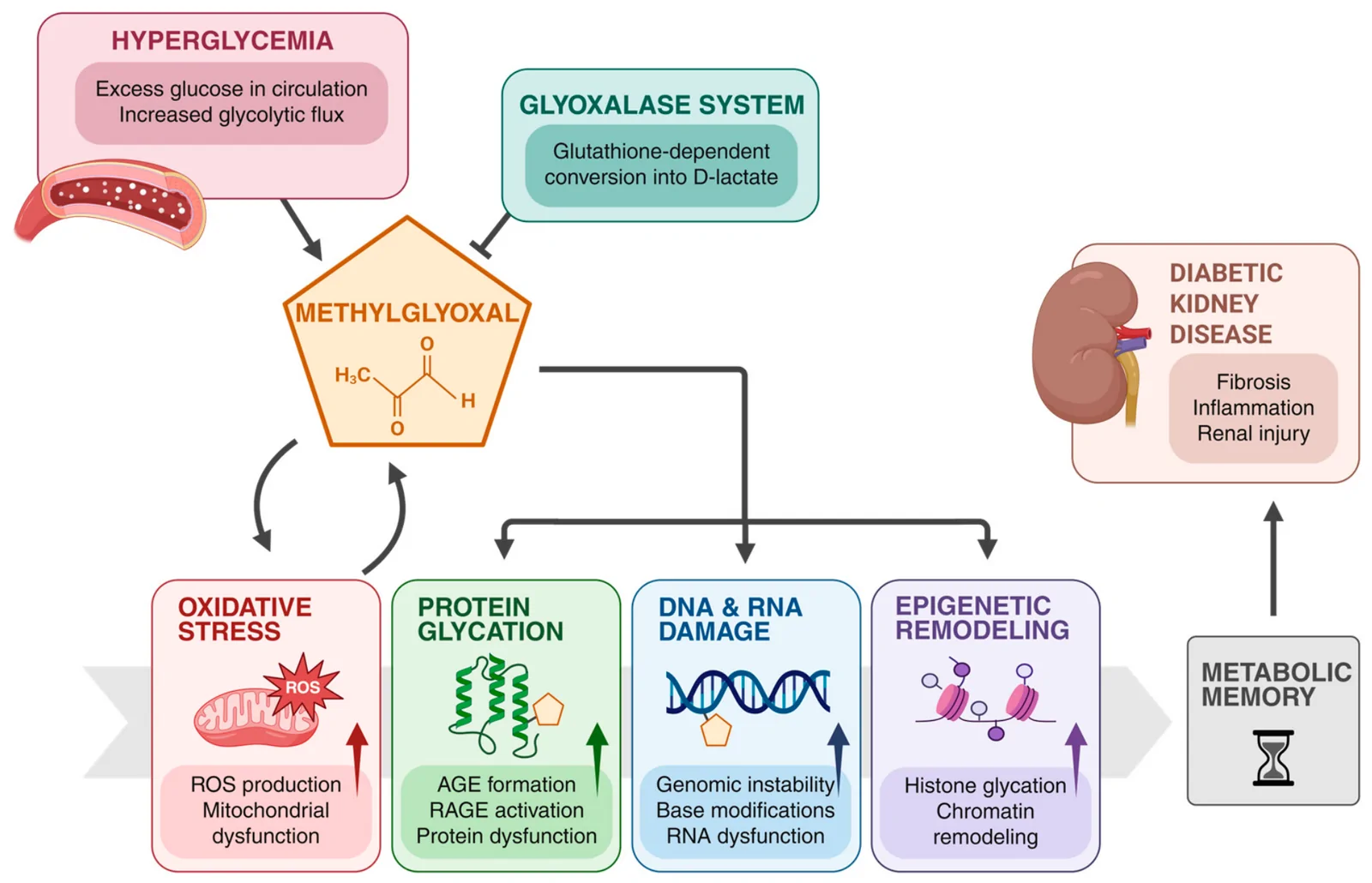
Schematic overview of the proposed role of methylglyoxal in metabolic memory. Hyperglycemia increases methylglyoxal (MGO) formation, while the glyoxalase system limits its intracellular accumulation. MGO and oxidative stress form a positive feedback loop, promoting persistent protein, DNA/RNA and epigenetic modifications. Together, these molecular alterations may contribute to metabolic memory and the development and progression of DKD. The ROS–MGO positive feedback loop represents a potentially self-sustaining process, whereas the persistence of molecular alterations after normalization of both glucose and MGO represents the proposed molecular memory component. Created in BioRender. Stelzer, M.A.K. (2026) https://BioRender.com/1y5hbso.

**Figure 2:**
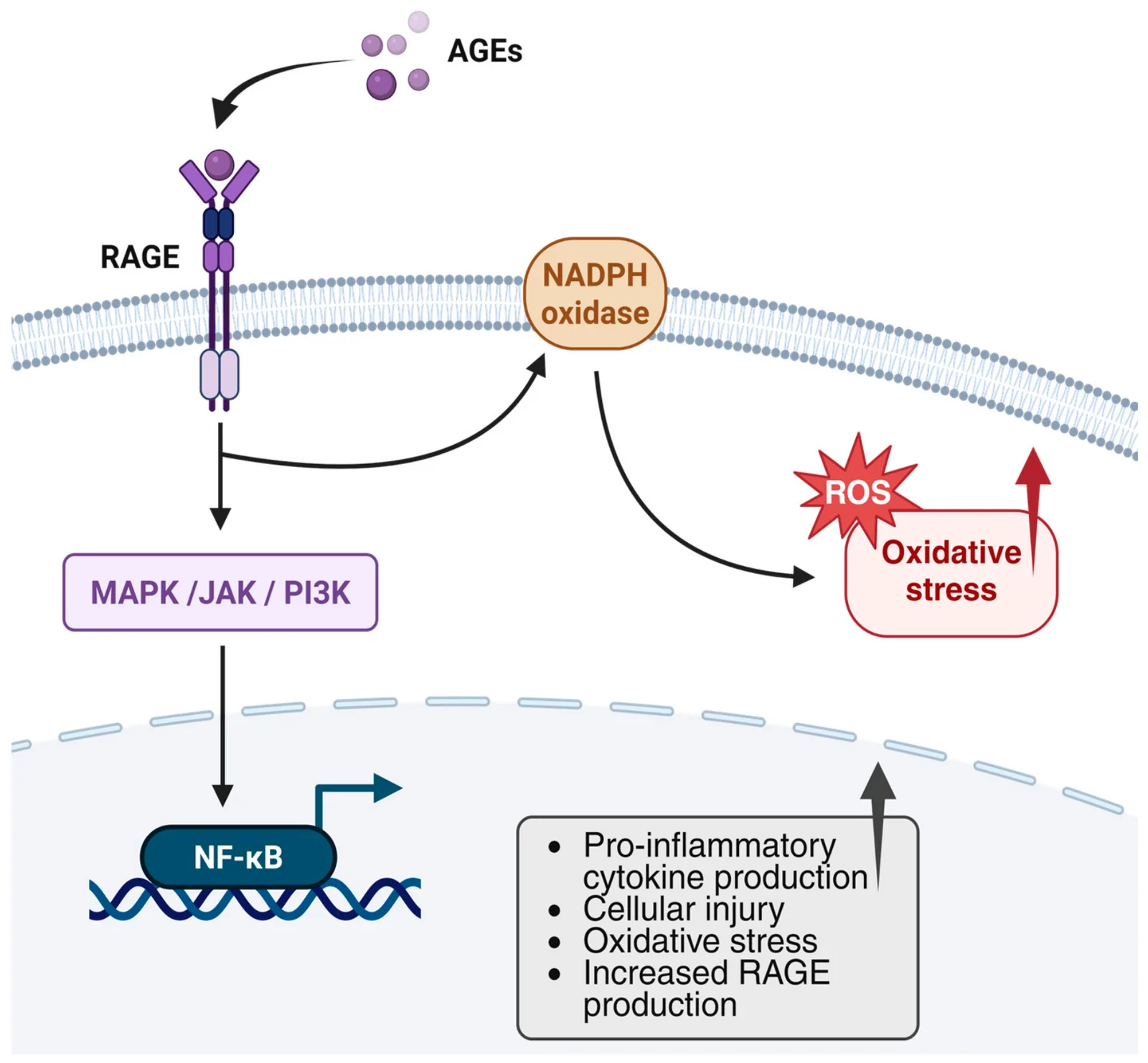
AGE binding to RAGE results in the activation of MAPK, JAK and PI3K signaling pathways. This results in the activation of the NF-κB pathway, consequently leading to an inflammatory response. RAGE activation also results in ROS production through NADPH oxidase activation. Created in BioRender. Stelzer, M.A.K. (2026) https://BioRender.com/0qper4k.

**Figure 3:**
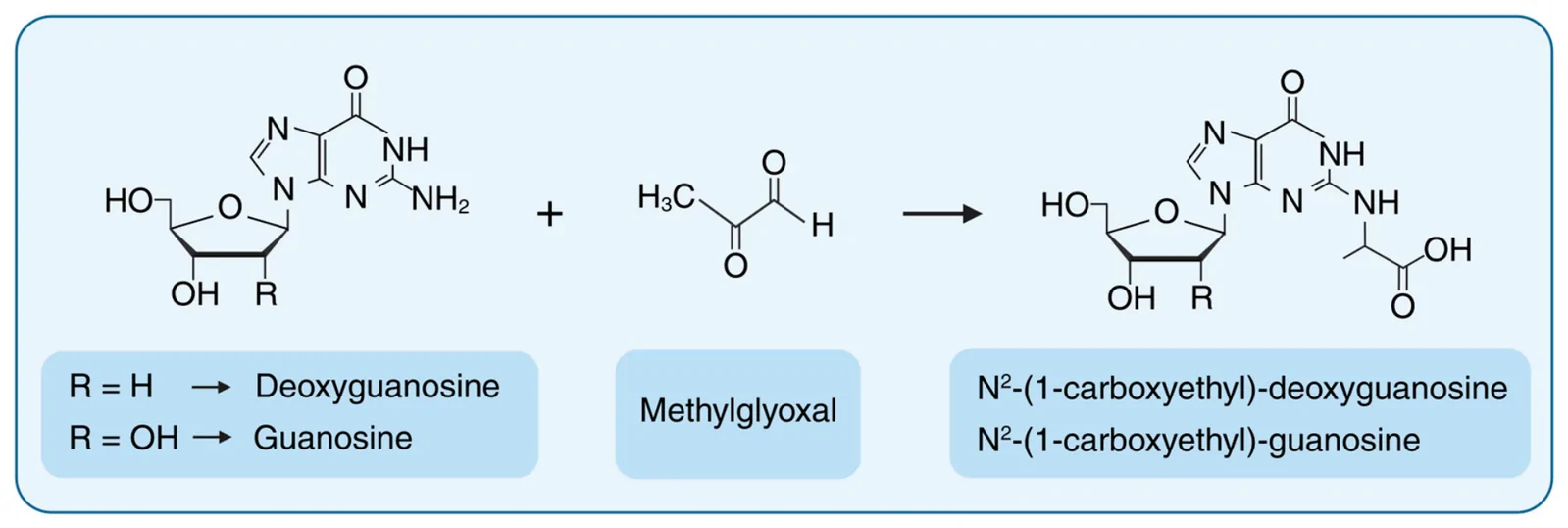
Methylglyoxal reacts with guanine residues to form N^2^-(1-carboxyethyl) guanine adducts. The 2′ substituent of the ribose distinguishes the DNA and RNA forms: R = H represents deoxyguanosine and the resulting CEdG adduct, whereas R = OH represents guanosine and the resulting CEG adduct. Created in BioRender. Stelzer, M.A.K. (2026) https://BioRender.com/5smk9zv.

**Figure 4:**
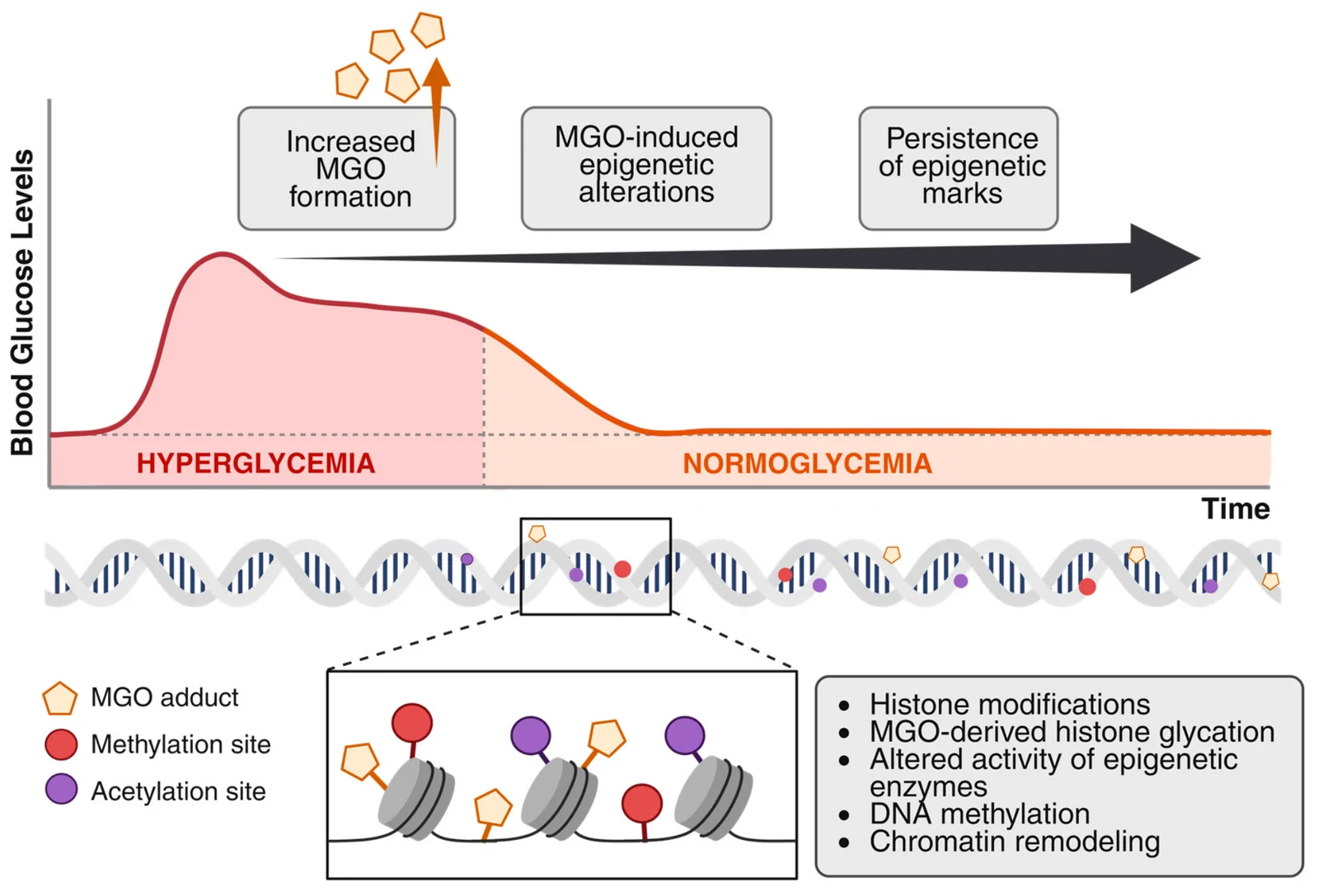
During hyperglycemia, increased blood glucose levels promote MGO formation, which can induce epigenetic alterations, including histone modifications, MGO-derived histone glycation, altered epigenetic enzyme activity, DNA methylation and chromatin remodeling. Following normalization of blood glucose levels, some of these epigenetic marks may persist and contribute to longer-lasting changes in gene regulation. Created in BioRender. Stelzer, M.A.K. (2026) https://BioRender.com/nupb5kh.

## Data Availability

No new data were created or analyzed in this study. Data sharing is not applicable to this article.

## Abbreviations

The following abbreviations are used in this manuscript:

DKD	Diabetic kidney disease
MGO	Methylglyoxal
T1D	Type 1 diabetes
T2D	Type 2 diabetes
OGTT	Oral glucose tolerance test
AGE	Advanced glycation end-product
ROS	Reactive oxygen species
GLO	Glyoxalase
MG-H1	Methylglyoxal-derived hydroimidazolone
CEdG	N^2^-(1-carboxyethyl)-2′-deoxyguanosine
Trx2	Thioredoxin 2
eGFR	Estimated glomerular filtration rate
CEL	Nε-(carboxyethyl)-lysine
ArgPyr	Argpyrimidine
RAGE	Receptor for advanced glycation end-products
NF-кB	Nuclear Factor kappa B
ECM	Extracellular matrix
dG	Deoxyguanosine
ATM	Ataxia–Telangiectasia mutated
nDNA	Nuclear DNA
mtDNA	Mitochondrial DNA
CEG	N^2^-(1-carboxyethyl)-guanosine
rNTP	Ribonucleotide triphosphate
dNTP	Deoxyribonucleoside triphosphate
ncRNA	Non-coding RNA
H3K4	Histone 3 lysine 4
Nrf2	Nuclear factor erythroid 2-related factor 2
EZH2	Enhancer of zeste homolog 2
H3K27	Histone 3 lysine 27
